# Common drug review recommendations for orphan drugs in Canada: basis of recommendations and comparison with similar reviews in Quebec, Australia, Scotland and New Zealand

**DOI:** 10.1186/s13023-018-0759-9

**Published:** 2018-01-30

**Authors:** John I. McCormick, L. Diana Berescu, Nabil Tadros

**Affiliations:** 1 McKesson Specialty Health, 6355 Viscount Road, Mississauga, ON L4V 1W2 Canada; 2 McKesson Specialty Health, 4705 Dobrin Street, Quebec, Saint-Laurent H4R 2P7 Canada

**Keywords:** Common Drug Review, Orphan drugs, Canadian Agency for Drugs and Technologies in Health, Reimbursement, Cost-utility

## Abstract

**Background:**

Public payer reimbursement for non-oncology drugs in Canada, including orphan drugs, is based on recommendations by the Common Drug Review (CDR) (with the exception of Quebec). CDR has been criticized for negative recommendations for orphan drugs and contributing to delays in patient access to these drugs. However, it is unclear how CDR makes recommendations for orphan drugs and the role clinical and economic factors play in decision making. The objective of the present study was to analyze the basis for CDR orphan drug recommendations and to compare recommendations to those in other jurisdictions.

**Methods:**

A list of orphan drugs reviewed by CDR (between 2004 and 2017) was compiled and final recommendations (list/do not list) assessed. The basis of each recommendation was categorized as clinical only, price only or combined clinical and price factors, based on the ranking of clinical and price parameters in recommendation summaries. The reimbursement status of the same drugs was determined in Quebec and other jurisdictions and level of agreement with CDR decisions assessed using a kappa analysis.

**Results:**

Sixty eight orphan drug submissions were identified in the CDR database. Clinical, clinical and price and price parameters were the basis of 48.5%, 44.1% and 7.4% of the reviews, respectively, and corresponding positive recommendation rates were 45.5%, 86.7% and 40.0% (*p* = 0.0008); overall positive recommendation rate was 63.2%. Positive recommendation rate increased from 50.0% for drugs reviewed between 2004 and 2009 to 86.7% in 2016; however, 84.6% of the latter were conditional on a price reduction. Of the drugs reviewed by CDR, 80.9%, 88.2%, 80.9% and 58.8% were reviewed for the same indications by health technology assessment agencies in Quebec, Scotland, Australia and New Zealand, respectively, with positive listing rates ranging from 60.0% (Quebec) to 92.7% (Australia) with fair (kappa coefficient 0.3307) to poor (kappa coefficient 0.0611) agreement with CDR in listing decisions, respectively.

**Conclusions:**

The positive CDR recommendation rate for orphan drugs was highest when clinical and price parameters supported the assessment. Over time there has been an increase in CDR positive recommendation rates for orphan drugs, although most are conditional on a price reduction. It is unclear if this change in CDR recommendations will impact equitable and timely access to orphan drugs across Canada.

**Electronic supplementary material:**

The online version of this article (10.1186/s13023-018-0759-9) contains supplementary material, which is available to authorized users.

## Background

Until recently Health Canada was working on an orphan drug framework with a proposed definition of a rare or orphan disease as one that affects < 1in 2000 persons, a definition aligned to that used in the European Union [1, 2]. Approximately 7000 such diseases have been identified and it is estimated that 1 in 12 Canadians, or about 2.8 million individuals, may be living with a rare disease [1, 3]. Therefore, although rare diseases have a low individual prevalence, collectively they can impart a large societal clinical and economic burden.

The reimbursement of orphan drugs presents unique challenges for both pharmaceutical manufacturers and reimbursement decision makers. The limited number of available patients means small clinical trial populations making it difficult to obtain valid comparative efficacy data [4, 5]. This creates issues for reimbursement authorities who may have to make decisions under conditions of uncertainty, particularly at the time of market authorization. In addition, for pharmaceutical manufacturers the small number of patients makes it difficult to recover drug development costs, unless the drugs are premium priced. This high cost in turn creates further issues for payers where application of traditional measures of efficacy and cost defining acceptable criteria for reimbursement may disqualify drugs for orphan diseases [4–6]. The overall result is that patients can have limited access to this class of drug. Several countries have defined policies that provide incentives to pharmaceutical companies to develop drugs for orphan diseases and reimbursement policies designed specifically to enhance patient access to these drugs [7–11]. However, legislative steps regarding access to orphan drugs in Canada have evolved slowly [12, 13]. In October 2012 the Canadian government took steps to address orphan diseases with the creation of a framework to facilitate research and authorization of new drugs for rare diseases [14, 15]. However, the final framework was never released and recently (October 2017) all reference to the framework has been removed from the Health Canada web site [16]. Instead, Health Canada is now working on an overall regulatory review of drugs and devices, including drugs for rare diseases, although few details of the impending changes to regulatory policies have been released [16].

In the absence of a national policy or a distinct regulatory and reimbursement pathway, orphan drugs go through the same review process as any other drug in Canada [17]. Public payer reimbursement for non-oncology drugs in Canada, with the exception of Quebec, is based on recommendations issued by the Common Drug Review (CDR) which was established in 2002 to provide a single national review process for approved drugs [17, 18]. In response to a submission, CDR review the clinical and economic evidence relating to the drug and prepares summary reports for its expert committee, the Canadian Drug Expert Committee (CDEC), who then reviews the evidence and issues a non-binding positive or negative recommendation for listing to participating publicly funded drug plans [17–19]. Each jurisdiction then decides whether or not it wants to participate in collective product listing negotiations through the pan-Canadian Pharmaceutical Alliance (pCPA) and ultimately if it wants to list the product [20]. Oncology drug submissions are reviewed by the pan-Canadian Oncology Drug Review (pCODR) through a similar process [21]. Both CDR and pCODR are part of the Canadian Agency for Drugs and Technologies in Health (CADTH).

CDR has been criticized for low rates of positive listing recommendations for new drugs [19, 22]. An assessment of all CDR reviews between 2003 and 2009 [19] found that of 138 final recommendations, 52% had a positive listing recommendation, a rate similar to that in Australia (54%) but considerably lower than the 87% rate reported for the United Kingdom [19, 23]. The CDR has also been criticized for negative recommendations for drugs used to treat orphan diseases and contributing to delays in patient access to these drugs in Canada [6, 24]. Although the organization has no specific policies regarding orphan drug technology assessments, an updated CDR framework published in March 2016 does provide guidance on the evaluation process for drugs that meet a significant unmet need for a rare condition, defined, on a population basis, in terms of the rarity of the condition and the absence of alternative treatments [25, 26]. However, it is still unclear how the CDR makes recommendations for orphan drugs and the role clinical and economic factors play in driving these decisions, or if the new framework has had any impact on how these drugs are reviewed. In addition, although it is inferred that in countries with specific orphan drug policies patients may have improved access to orphan drugs compared to Canada, there is limited evidence that this is the case. In the current study we have reviewed CDR recommendations for orphan drugs, defined the parameters involved in decision making, and compared recommendations with those made in Scotland, Australia and New Zealand. These countries were chosen because they have differing approaches to orphan drug reimbursement and the information on status of orphan submissions is easily accessible. In addition, we compare CDR recommendations with those made in Quebec by the Institut national d’excellence en santé et en services sociaux (INESSS), since this province maintains its own drug review process independent of the CDR.

## Methods

### Orphan drug dataset

A list of orphan drugs was obtained from the Orphanet web site [27]. Orphanet is an online reference source for information on rare diseases and orphan drugs and the web site provides a comprehensive list of drugs granted an orphan designation in Europe [27]. The Orphanet portal was initially a component of the Canadian government initiatives designed to support research and approval of these drugs [2]. The Federal Drug Administration (FDA) list of drugs with orphan designations in the USA was also used to augment the list of drugs included in the study [28]. A list of the orphan drugs included in the study, and their indications, is provided in Table S1 in the Additional file 1.

### Data collection: CDR recommendations

Publicly available CDR recommendations were reviewed for each identified orphan drug (review period was January 2004 to October 2017) and the following information was extracted from the Recommendation and Reasons file [29]: date of CDR recommendation, generic and brand name of drug, drug indication, type of recommendation, basis of decision for recommendation, incremental cost-utility ratio (ICUR) (if provided) and whether or not recommendation was based on a request for reconsideration [29]. The latter is a process where participating drug plans or the manufacturer can issue a request for reconsideration following an initially negative draft CDR recommendation; this initiates an additional CDR review and issuance of a final recommendation [18]. Only final CDR recommendations and the date of the decision were included in the analysis; if a drug had more than one submission for the same indication, only the result of the most recent submission was recorded. In most cases, ICUR values recorded were those provided by the manufacturer; exceptions to this rule when CDR determined the ICUR are described in table footnotes. If the ICUR was reported as confidential with a CDR comment that the ratio exceeded acceptable values, it was assumed that the ICUR was >$100,000/quality adjusted life-year (QALY).

CDR recommendation categories have changed over time and the current options (March 2016) are: reimburse; reimburse with clinical criteria and/or conditions; do not reimburse [25]. However, over the timeframe of the study, CDR recommendation options were: do not list; do not list at the submitted price; list with clinical criteria and/or conditions; list [18]. Nevertheless, for this analysis recommendations were recorded only as list or do not list and whether the listing recommendation was conditional on a reduction in price.

The basis of decisions for recommendations was categorized in a similar manner to that defined in a prior study assessing all CDR recommendations [19]. In the majority of CDR reviews, reasons for recommendation are listed and Rocchi et al. [19] hypothesized that they were listed in hierarchical level of importance for at least the first two factors (see Supplemental Digital Content in reference [19]). On this basis, a primary reason for recommendation was defined, based on the first two reasons listed, in the following manner:

- Clinical only: First two reasons cite clinical factors
- Price only: First reason cites price
- Clinical and price: First reason cites a clinical factor, second reason cites price

An identical definition was used in the current study to describe the basis of the decision for each orphan drug recommendation.

### Data collection: International organizations

To quantify how CDR recommendations for orphan drugs compare with other public payers, the reimbursement recommendations for the same drugs and indications were reviewed in Scotland, Australia, New Zealand and Quebec.

In Scotland drug submissions are reviewed by the Scottish Medicines Consortium (SMC) whose task, similar to that of the National Institute for Health and Care Excellence (NICE) in England, is to provide advice to National Health Service (NHS) boards across Scotland on the clinical and cost-effectiveness of newly licensed medicines [30]. SMC has adapted the definition of orphan drugs provided by the European Medicines Agency (EMA) and has recently defined procedures specifically for the evaluation of end-of-life drugs and drugs used to treat very rare conditions [31]. As of May 2014, pharmaceutical companies can request that SMC convenes a Patient and Clinical Engagement (PACE) group for drugs in this class allowing patient groups and clinicians a stronger voice in decision making [31].

In Australia, drug-funding decisions are made through the Pharmaceutical Benefits Advisory Committee (PBAC) [32]. Although PBAC recognizes the importance of the treatment of rare diseases (defined as a disease with a prevalence of ≤ 2000 individuals in Australia), there are no specific evaluation procedures for drugs in this category [32]. However, patients can get access to orphan drugs through the Life Saving Drugs Program (LSDP), which provides funding for drugs that may have been rejected by PBAC because of an unacceptable cost-effectiveness ratio. Drugs eligible for the LSDP are defined by a number of criteria including disease rarity, impact of the disease on patient life-expectancy and current treatment alternatives [33–35].

Drug funding decisions in New Zealand are made through the Pharmaceutical Management Agency (PHARMAC) with input from the Pharmacology and Therapeutics Advisory Committee (PTAC) [36, 37]. Historically there were no specific policies regarding access to orphan drugs in New Zealand. However, in 2014 PHARMAC reviewed their procedures for funding orphan drugs and introduced a trial contestable fund and bidding process for suppliers to encourage more competitive pricing [38, 39]. To support this process PHARMAC has allocated $25 million over 5 years for orphan drug funding [40].

Currently there is no specific policy regarding reimbursement of orphan drugs in Quebec, but INESSS has initiated a review of how rare diseases are managed in other countries, which led to a recommendation that similar policies be adapted in Quebec and that collaboration through participation in Orphanet be prioritized [41, 42].

To compare CDR recommendations for the selected orphan drugs, web sites with information on listings for the same drugs and indications in Scotland [43], Australia [44], New Zealand [40, 45] and Quebec [46, 47] were searched. If a specific drug had been evaluated in other jurisdictions, decisions were recorded as either not listed or listed; in the case of the latter, the specific listing criteria were not recorded. For Australia, drugs that were rejected and deferred by PBAC were included in the not listed category. Decisions for New Zealand include drugs approved for funding as a result of a test of a commercial process aimed at improving patient access to drugs for rare diseases [40].

### Statistical analysis

Recommendation rates for CDR and the additional jurisdictions were reported as number and percent of total reviews with a positive recommendation. Fisher’s exact test was used to test for differences in CDR recommendation rate based on clinical, clinical and price and price parameters. It was also used to test recommendation rate difference in submissions with and without an ICUR. Concordance in recommendations between CDR and the other jurisdictions was expressed as the proportion of reviews with identical decisions (to list or not to list). The level of agreement between jurisdictions was determined using a kappa analysis. In this analysis a kappa value of ≤0.2 indicates a poor level of agreement while values ranging from 0.21–0.40 to 0.81–1.0 define levels of fair to very good agreement, respectively [48].

## Results

In the review of CDR data, 68 submissions were identified for 59 orphan drugs with nine drugs submitted for two indications. Details of the identified drugs, their indications, final recommendations, the reasons for the recommendation and ICURs provided are summarized in Table S1 in Additional file 1. The overall information on the submissions is summarized in Table 1. Of the 68 submissions, CDR recommended that 43 (63.2%) of the drugs be listed, most with clinical criteria and/or conditions defining their use. There was a significant variation in recommendation rates when submissions were categorized in terms of the parameters driving CDR decisions. The majority of decisions (48.5%) were based on clinical parameters with 44.1% and 7.4% based on clinical and price and price only parameters, respectively. However, the positive recommendation rate for decisions based on clinical and price parameters (86.7%) was significantly higher than rates based on clinical (45.5%; *p* = 0.0012) or price (40.0%; *p* = 0.0438) only parameters (Table 1).

**Table 1:** Summary of CDR^a^ recommendations for orphan drugs

Category	Number	Positive recommendations (n (%))^b^
Total number of submissions	68	43 (63.2%)
Recommendations based on clinical parameters only (n (%))^c^	33 (48.5%)	15 (45.5%)
Recommendations based on clinical/price parameters (n (%))^c^	30 (44.1%)	26 (86.7%)
Recommendations based on price parameters only (n (%))^c^	5 (7.4%)	2 (40.0%)
Submissions over different time periods:		
2004–2009	22 (32.4%)	11 (50.0%)
Number of positive recommendations with a conditional price reduction^e^		0 (0.0%)^d^
2010–2011	7 (10.3%)	3 (42.9%)
Number of positive recommendations with a conditional price reduction^e^		2 (66.7%)^d^
2012–2013	12 (17.6%)	8 (66.7%)
Number of positive recommendations with a conditional price reduction^e^		2 (25.0%)^d^
2014–2015	12 (17.6%)	8 (66.7%)
Number of positive recommendations with a conditional price reduction^e^		6 (75.0%)^d^
2016	15 (22.1%)	13 (86.7%)
Number of positive recommendations with a conditional price reduction^e^		11 (84.6%)^d^
Recommendations with no ICUR (n(%))^f^	36 (52.9%)	21 (58.3%)
Recommendations with an ICUR (n(%))^g^	32 (39.2%)	22 (68.8%)
Recommendations based on a reconsideration	25 (36.8%)	8 (32.0%)

^a^Abbreviations are: *CDR* Common Drug Review, *ICUR* incremental cost-utility ratio, *QALY* quality adjusted life-year

^b^Refers to a recommendation for listing regardless of the specific type of listing; percentage is based on the total number of reviews in each category

^c^Categories based on the sequence of factors listed in the reasons for recommendation in CDR reviews (see Methods)

^d^Percent based on the total number of positive recommendations over each time period

^e^Conditions of recommendation include a substantial reduction in price or that price should not exceed the cost of a comparator therapy on drug plan formularies

^f^Includes recommendations which provided a cost per life year gained (*n* = 2) but excludes recommendations where the manufacturer requested that the ratio remain confidential (*n* = 2)

^g^Includes submissions where manufacturers requested that ICUR remain confidential (n = 2) but excludes assessment of cost/life year gained (n = 2)

Positive recommendation rates increased dramatically between 2004 and 2016. Over the earlier period (2004 to 2009) only 50% of submissions (*n* = 22) resulted in a recommendation for listing but in 2016 this increased to 86.7% (based on 15 submissions) (Table 1). However, in 2016 of the 13 recommendations for listing, 11 (84.6%) were conditional on a substantial reduction in price, or a reduction in drug price to that of a comparator drug for the same indication. The frequency of use of this conditional price listing by CDR increased 3.4-fold between 2012 and 2013 (25.0% of recommendations had a cost limitation) and 2016.

The majority of submissions (52.9%) did not report an ICUR and economic evaluations appeared to be based on statements regarding monthly/annual costs of therapy although the type of economic evaluation applied was not always clear. Submissions including an ICUR had a higher likelihood of positive recommendation (68.8% vs. 58.3% (Table 1)) but this difference was not statistically significant (*p* = 0.4535); approval rates were highest when ratios were in the range of $50,000 - $100,000/QALY (Table 2). However, as shown in Table 2, there was a trend for increased positive recommendations for drugs with a high ICUR in 2016. There were nine submissions in 2016 for drugs with ICURs > $100,000/QALY (mean and median ICURs of the nine drugs were $958,443/QALY and $488,182/QALY, respectively) and the positive recommendation rate was 77.8%, all conditional on a reduction in price. In contrast, the positive recommendation rate for drugs with ICURs > $100,000/QALY prior to 2016 was only 42.9% (Table 2).

**Table 2:** Positive orphan drug recommendation rates for different ICUR threshold values

Cost-utility threshold	Number of submissions with cost-utility values in this range (N)^a^	Positive recommendation rate (%)
≤ $50,000/QALY^b^	6^c^	66.7%
$50,000 - $100,000/QALY	11^d^	72.7%
> $100,000/QALY^e^	16	62.5%
Prior to 2016	7	42.9%
2016	9	77.8%^f^

^a^For submissions with a range of values provided in the CDR recommendation, the lower value of the range was used for this analysis

^b^Abbreviations are: *QALY* quality adjusted life-year, *NHL* Non-Hodgkin’s Lymphoma, *MM* Multiple Myeloma, *ICUR* incremental cost-utility ratio

^c^Includes deferiprone (iron overload) which was dominant and stiripentol (Dravet Syndrome) where ccost-utility ratio was $50,122/QALY

^d^Ratios for plerixafor (NHL (< $50,000/QALY) and MM ($50,000–$100,000/QALY)) and sunitinib (metastatic renal cell carcinoma and gastrointestinal stromal tumor both in the $50,000–$100,000/QALY category) are included twice in the calculations

^e^This category also includes drugs with a “confidential” recommendation (*n* = 2) based on the assumption that their ICURs were > $100,000/QALY

^f^All of the positive recommendations in this category had a conditional price reduction

The new CADTH framework for evaluating drugs was published in March 2016 [25, 26]. Table 3 summarizes information in CDR reviews on orphan drugs with high ICURs that were rejected prior to the release of the March 2016 framework and compares them with comments on drugs recommended after the framework was introduced. These CDR comments can provide information on why specific drugs were or were not recommended for reimbursement. In the examples shown (Table 3), prior to March 2016, CDR questioned the clinical relevance of outcomes assessed in clinical trials and this, together with the high ICURs, led to the negative recommendation. In addition, over this earlier period patient input appeared to have little influence on decision making. However, after March 2016 there was a change in the tone of CDR comments; now, although there were ongoing concerns about the relevance of clinical trial outcomes, the drugs were recommended for reimbursement, despite their high ICURs, but with a conditional price reduction and defined clinical criteria. In some cases CDR suggested the specific price reduction required to reduce the ICUR to approximately $100,000/QALY (e.g., a 97% suggested reduction in price for elosulfase alfa; Table 3). In addition, in the post-March 2016 reviews, patient input appeared to be more comprehensive and may have played a larger role in the final decision.

**Table 3:** CDR^a^ comments on recommendations for orphan drugs with high ICURs

Drug	Disease	Recommendation (Date of decision)	ICUR^b^	Patient input	CDR comments
Eculizumab	Paroxysmal nocturnal hemoglobinuria	Do not list at the submitted price (19–02-2010)	$500,000 to $2.4 million/QALY (vs. BSC)	No reference to patient input	Double blind RCT (*n* = 87 patients) showed a significant reduction in hemolysis and improvement in QoL with eculizumab (versus placebo) but was not designed to assess effect on survival or thrombotic events, a prognostic factor for survival. CDR concluded that the drug was not cost-effective
Eltrombopag olamine	Chronic immune thrombocytopenic purpura (ITP)	Do not list (24/10/2011)	Confidential^c^	No patient groups responded to the CDR call for input	Data from three RCTs (*n* = 429) was reviewed but only one trial (*n* = 197) was discussed (others were excluded because of a limited study duration). In the study, the percentage of patients achieving the primary outcome (platelet response) was significantly higher with eltrombopag. However, CDR considered this outcome less clinically relevant than bleeding events and noted that there were no RCTs comparing eltrombopag with individual comparator ITP therapies
Tolvaptan	Autosomal dominant polycystic kidney disease (ADPKD)	Do not list (24/02/2016)	$244,402/QALY (vs. BSC)	Noted the burden of ADPKD on patients and caregivers and stressed that only tolvaptan has been approved for treatment of ADPKD. Patients expect tolvaptan toprolong their lives and improve QoL but also noted the challenges of using tolvaptan (large daily fluid intake, increased urination, dry mouth and thirst)	One Phase III, RCT (*n* = 1445) showed that tolvaptan significantly delayed clinical progression. However, CDR was concerned about the importance of these outcomes and the extent to which clinical changes are maintained over a patient’s lifetime and potential safety issues associated with the drug. NICE have recommended tolvaptan for rapidly progressing disease but CDR concluded that at present identifying such patients would create challenges for drug plans to consistently implement and the cost-effectiveness of the drug in these populations could not be evaluated, based on available data
Revised CADTH Framework released in March 2016					
Asfotase alfa	Pediatric-onset hypophosphatasia (HPP)	List with criteria and conditions^d^ (23/03/2016)	$2,698,950/QALY (vs. BSC)	Identified a substantial unmet need in treatment of HPP. Details of the clinical impact of the disease were provided and the particular burden oncaregivers emphasized. Patients and caregivers noted that this is the first therapy approved for HPP and that they would be willing to accept extensive side effects if overall QoL was improved	Two open-labelled Phase II studies (*n* = 72) and one extension study demonstrated improvement in skeletal development and patients appeared to have a lower rate of mortality compared with the rate of HPP mortality from natural disease history data. Because of the heterogeneous patient population, CDR recommended that drug plans and clinical experts establish case-by-case evaluation criteria for initiation and continuation of therapy. CDR considered asfotase alfa not to be cost-effective at the submitted price
Elosulfase alfa	Mucopolysaccharidosis IVA	List with criteria and conditions^d^ (20/05/2016)	$1,720,127/QALY (vs. BSC)	Patients expressed a desire to see disease progression stabilized or slowed. Patients receiving elosulfase alfa reported improvements in endurance and stabilization of condition and did not report any major AEs	One double-blind RCT (*n* = 177) demonstrated significant improvement in six-minute walk test vs. placebo. However, CDR noted uncertainties regarding relevance and validity of the walk test as an outcome and recommended a manufacturer sponsored international registry to collect clinical data. In addition, CDR recommended that goals of therapy be defined on a case-by-case basis and reassessed after 1 year of therapy. Uncertainty was reported regarding true cost-effectiveness and CDR noted that a price reduction of 97% would be required for the ICUR to approach $100,000/QALY
Canakinumab	Systemic juvenile idiopathic arthritis (sJIA)	List with criteria and conditions^d^ (17/06/2016)	$824,000/QALY (vs. BSC)	Inability to perform daily routine activities imparts a severe psychological burden on patients and their caregivers. Patient groups noted that responses to treatment options available can vary significantly	Two RCTs (*n* = 184) demonstrated that canakinumab improved patient QoL, decreased pain and improved function compared to placebo. There are no direct comparisons of canakinumab to other biologic therapies in sJIA but indirect comparisons suggest that it has similar efficacy. One of the criteria of reimbursement is that treatment be discontinued if there is no improvement after day 15. CDR determined that canakinumab is 10 to 15 times higher in price than other sJIA treatments and a reduction in price of approximately 90% would be required to make it a cost-effective option vs. tocilizumab; condition of reimbursement is that drug price should not exceed drug plan cost of tocilizumab

^a^Abbreviations are: *CDR* Common Drug Review, *QALY* quality adjusted life-year, *BSC* Best supportive care, *ICUR* incremental cost-utility ratio, *QoL* quality of life, *RCT* randomized controlled trial, *NICE* National Institute for Health and Care Excellence, *AE* adverse event

^b^Cost utility ratios are those provided by the manufacturer except for eculizumab and asfotase alfa where the ratio was determined by the Common Drug Review, based on the manufacturer’s data

^c^Submissions where cost utility ratios were confidential; CDR noted that ratios greatly exceeded conventional standards for cost-effectiveness

^d^For these recommendations CDR included a condition of a substantial reduction in price or a drug cost that should not exceed the drug plan cost of a comparator therapy

Twenty five (36.8%) of the recommendations were based on a reconsideration but this had little impact on the final decision and only eight drugs (32.0%) were recommended for listing following this additional review (Table 1).

As shown in Table 4, of the 68 CDR drug submissions identified, 55 (80.9%), 60 (88.2%), 55 (80.9%) and 40 (58.8%) were also reviewed by INESSS, SMC, PBAC and PHARMAC, respectively. Positive listing rates for reviewed drugs were similar in all jurisdictions except Australia where 92.7% of the drugs were listed, although 11 (20.0%) of the drugs were only available through the LSDP; in the absence of LSDP availability, the recommendation rate in Australia decreased to 72.7%. Additional details on listing decisions in each jurisdiction are provided in Table S2, Additional file 1.

**Table 4:** Comparison of recommendation rates for the selected drugs in Canada (CDR),^a^ Quebec (INESSS), Scotland (SMC), Australia (PBAC) and New Zealand (PHARMAC)

Orphan drug status	CDR	INESSS	SMC	PBAC^b^	PHARMAC
Number of drugs reviewed (N)	68	55	60	55	40
Drugs with a positive listing recommendation (N(%))	43 (63.2%)	33 (60.0%)	38 (63.3%)	51 (92.7%)^b^	27 (67.5%)
Degree of concordance with CDR recommendations (%)	–	69.1%	70.0%	65.5%	62.5%
Kappa coefficient	–	0.3307	0.3541	0.0611	0.1620

^a^Abbreviations are: *CDR* Common Drug Review, *INESSS* Institut national d’excellence en santé et en services sociaux, *SMC* Scottish Medicines Consortium, *PBAC* Pharmaceutical Benefits Advisory Committee, *PHARMAC* Pharmaceutical Management Agency

^b^Eleven drugs were funded through the Life Saving Drugs Program

To assess the degree of concordance in recommendations with those of CDR, common drugs reviewed by jurisdictions were identified and the degree of identical recommendations (i.e., listed or not listed) assessed. As shown in Table 4, concordance with CDR recommendations ranged from 62.5% with PHARMAC to 70.0% for SMC. However, the kappa analysis, which assesses the level of agreement between jurisdictions, revealed only fair agreement in decisions between CDR and INESSS and SMC with kappa coefficients of 0.3307 and 0.3541, respectively. In contrast, agreement in decisions between CDR and PBAC and PHARMAC was poor with a kappa coefficient < 0.20. Only 36.4% of the common drugs reviewed by all four agencies (*n* = 33) had the same recommendation.

## Discussion

### Clinical uncertainty and CDR recommendations

Using criteria previously defined to identify primary reasons for CDR recommendations [19], the majority of CDR orphan drug recommendations were based on the strength of clinical parameters alone but the subsequent positive recommendation rate was only 45.5% versus 86.7% for recommendations incorporating both clinical and price parameters (*p* = 0.0012). Therefore, there was a low probability of a positive recommendation when CDR decisions were driven by clinical outcomes and where drug cost was not a major factor in the evaluation. The relevance of clinical outcomes in driving CDR recommendations has been confirmed by Janoudi et al. [49] who in a recent review of 63 CDR orphan drug submissions (2004 to 2015) reported that the major reason for a negative reimbursement recommendation was lack of clinical effectiveness (38.5% of negative recommendations vs. only 7.7% for high cost-effectiveness/high cost). Clinical uncertainty has also been identified as a significant predictor of a do not list recommendation in all CDR reviews [19]. For most orphan drugs there is a degree of uncertainty regarding efficacy, effectiveness and safety [50]. Small potential patient numbers for clinical trials, reliance on surrogate outcomes, clinical trial periods that may not reflect disease duration, heterogeneity in patient response and unclear comparator therapies all contribute to this clinical uncertainty and compromise HTA assessments [50]. However, the March 2016 CADTH framework acknowledges that there may be limitations in clinical data for orphan drugs and that normal review procedures may have to be modified to address these limitations [25]. Although the present study presents historical data indicating that clinical uncertainty was a significant factor in rejecting orphan drugs for reimbursement, the data also suggest that the guidance provided in the revised CADTH framework has led to changes in how CDR reviews orphan drugs [25]. This is most apparent in the dramatic increase in positive recommendations in 2016 (86.7% in 2016 vs. 48.3% between 2004 and 2011). The overall recommendation rate over the complete assessment period (2004 to 2016) was 63.2%, a rate elevated by the more frequent listing recommendations in 2016. This compares with a CDR orphan drug recommendation rate of 55.4% (between 2004 and 2015) estimated from data reported by Janoudi et al. [49] when their data was expressed in the same way as in the present study (i.e., only including the most recent submission in the analysis for drugs with re-submissions). Overall CDR positive recommendation rates have been estimated at 52% (for the period 2003 to 2009) indicating that at least prior to 2016, orphan drugs were recommended for listing at a similar rate to other drugs [19].

A recent change in how CDR reviews orphan drugs was also apparent in CDR drug review comments. Whereas prior to the release of the 2016 framework, uncertain clinical data would have increased the probability of a negative recommendation, that same uncertain clinical data may now be more acceptable with criteria defining evidence development over time, particularly if the drug meets an unmet clinical need. As shown in the post-framework reviews described here (Table 3), elements of the CADTH March 2016 framework [25] were apparent, including recommendations for starting and stopping rules, real-world evidence development and characteristics of the care setting. However, almost all the positive recommendations in 2016 for drugs with high ICURs were conditional on a substantial reduction in price because CDR did not consider the drugs cost-effective at the submitted price. In addition, most recommendations came with clinical criteria and/or conditions defining drug use. As a result of the new framework, CDR appears more willing to accept clinical uncertainty through future real-world evidence development but has applied substantial cost reduction conditions for reimbursement.

Despite these changes, it is currently unclear how this new approach to recommendations for orphan drugs will impact patient access in Canada. It has been suggested [20, 51] that CDR recommendations with conditional price adjustment may guide subsequent pCPA pricing negotiations but since the pCPA price negotiations are confidential it is difficult to determine the final impact of such CDR recommendations. The new 2016 CADTH framework may actually delay patient access since the evidence summarized here suggests that the time taken for the CDR review has only resulted in a conditional cost recommendation for most reviewed drugs, the application of which is directly under the control of the drug plan (i.e. negotiation of a reduction in the drug price and adjustment of drug plan budgets to accommodate use of the drug). In addition, in some cases provincial Ministries of Health have decided to review orphan drug clinical data for themselves, and have made independent decisions on listing [52–54]. This further suggests that the CDR (or pCODR) review of orphan drugs may be somewhat redundant, only delaying patient access to the drugs at the drug plan level where the final decision on listing is ultimately made.

### Provincial listing decisions

Although this study is based on CDR recommendations, there is no legal requirement for the different public health plans to accept the CDR recommendation, although a positive CDR recommendation is a strong predictor of subsequent provincial listing. For example, a recent study found that for drugs with a positive CDR recommendation, 93% were subsequently available in Ontario [52]. However, for orphan drugs there is variable agreement between CDR decisions and subsequent provincial listing. Allen et al. [53] in a comprehensive review of 174 medicine-indication pairs in CDR reports (2009 to 2015) found 78.9%, 81.1% and 78.8% agreement between CDR recommendations and listing decisions in Alberta, British Columbia and Ontario, respectively, for all drugs. However, analysis of the Allen et al. [53] data for orphan drugs (22 of the drugs were identified as orphan drugs) indicated only 59.1% and 63.6% agreement with CDR recommendations in Alberta and Ontario, respectively, but 86.4% agreement in British Columbia; only 36.4% of the drugs had the same recommended listing in all three provinces. In the same way, a negative CDR recommendation does not necessarily preclude provincial listing and in Ontario 50% of all drugs with a negative CDR recommendation were subsequently available in the province [52]. Of the 22 orphan drugs identified in the Allen et al. [53] study, 45.5% had a negative CDR recommendation, but despite this 60.0% were listed in Ontario. This heterogeneity in provincial listing decisions for orphan drugs in Canada has also been noted by Menon et al. [54] and emphasizes the unequitable national access to this drug class.

### Interagency listing agreement

The CDR orphan drug recommendation rate (63.2% overall) was similar to the rates determined in other jurisdictions, based on percent concordance, except for Australia where patients have greater access to orphan drugs, aided, in part, by the LSDP program [33]. However, the kappa analysis indicated little evidence of consistency in recommendations. Variation in HTA agency decisions has been noted by others and likely reflects varying interpretation of the validity and relevance of pharmacoeconomic evidence, local drug prices, variation in patient populations meeting clinical treatment criteria, effectiveness of potential market comparators and different interpretation of the same clinical trial data [17, 55]. The urgent issue facing many HTA agencies is how to apply cost and clinical criteria, initially defined for non-orphan drugs, to drugs for rare diseases so that patients can have equitable and affordable access to treatment. In many countries the solution is one of conditional orphan drug reimbursement where the “condition” can be based on a variety of factors including a drug price reduction, temporary reimbursement with clinical evidence development, higher willingness-to-pay thresholds, creation of special orphan drug funds or risk-sharing agreements [56, 57]. However, there is no consensus as to the most effective approach and the HTA environment is currently fragmented in terms of the review and reimbursement of orphan drugs, as shown by the poor agreement in listing decisions shown in this study [56, 57]. With regard to Canada, the latest trends at CADTH suggest that orphan drug manufacturers should be prepared for real-world evidence studies which may be required to demonstrate the clinical value of an orphan drug in real clinical practice.

Concordance of CDR and INESSS orphan drug recommendations, where drugs were evaluated within very similar healthcare systems, was 69.1% (kappa coefficient 0.3307), emphasizing the potential for variable interpretation of presumably the same country-specific clinical and economic data. This level of concordance between INESSS and CDR for orphan drugs is similar to the degree of concordance (64.0%) recently reported for all non-cancer drugs [58].

### Limitations

There are a number of limitations in the present study. The detailed analysis of CDR recommendations relied only on publically available information; any confidential information included in submissions was not available for this review. In addition, the reasons for recommendation were based on assumptions on the hierarchical level of importance in the listing of reasons [19]. There are limitations in applying a structured process to define a primary reason for a recommendation to a fluid CDR review procedure incorporating multiple factors. Nevertheless, in the absence of other methods to calibrate CDR review, this procedure does provide a tool that can be used to define the CDR recommendation process and may be particularly effective in comparative analyses. Given the new CADTH framework, and the recognition of the special characteristics of orphan drugs, the positive recommendation rate for decisions based on clinical parameters alone will likely increase from the 45.5% observed here because CADTH has defined procedures to address clinical uncertainty. In fact this may be already happening; data from all 68 indications showed that 48.5% of CDR recommendations were based on clinical parameters alone with a 45.5% recommendation rate, reflecting clinical uncertainty. In 2016 (15 submissions), 53.3% were assessed on the basis of clinical parameters alone but 75.0% of the recommendations were positive, albeit with conditional price reductions, suggesting more confidence in clinical data.

Finally, assessing an absolute positive recommendation rate for orphan drugs can be subjective, depending on which drugs were included in the review and the criteria used for their inclusion. In addition, only four HTA agencies were included for comparison which may have limited the generalizability of the results.

## Conclusions

The positive recommendation rate for CDR reviews of orphan drugs was highest when both clinical and price parameters formed the basis of the assessment. However, there was a change in CDR review policies in 2016, with a dramatic increase in the number of positive recommendations, the majority of which were conditional on a substantial reduction in drug price. This change in CDR recommendations is reflected in the expanded criteria and conditions of drug reimbursement outlined in CADTH’s March 2016 recommendation framework. However, it remains to be seen if this represents a permanent change in how CDR reviews orphan drugs and if this will result in wider and more timely provincial access for orphan drugs for patients throughout Canada.

## Additional file

Additional file 1: Table S1. Detailed analysis of CDR reviews of identified orphan drugs. **Table S2.** Comparison of recommendations in Canada (CDR), Quebec (INESSS), Scotland (SMC), Australia (PBAC) and New Zealand (PHARMAC) for the selected orphan drugs. (DOC 208 kb)
