# Supplementary material for: Common drug review recommendations for orphan drugs in Canada: basis of recommendations and comparison with similar reviews in Quebec, Australia, Scotland and New Zealand
Source: Orphanet J Rare Dis. 2018 Jan 30;13:27. doi: 10.1186/s13023-018-0759-9 (PMC5791218; doi:10.1186/s13023-018-0759-9)
Supplement: Additional file 1: Table S1. — Detailed analysis of CDR reviews of identified orphan drugs. Table S2. Comparison of recommendations in Canada (CDR), Quebec (INESSS), Scotland (SMC), Australia (PBAC) and New Zealand (PHARMAC) for the selected orphan drugs. (DOC 208 kb) [file 13023_2018_759_MOESM1_ESM.doc]

**Additional file 1**

**Table S1: Detailed analysis of CDR^1^ reviews of identified orphan drugs**

| **Drug** | **Brand name** | **Disease** | **Recommendation** | **Reason for recommendation^2^** | **Cost-utility ratio^3^** | **Date of recommendation**  **(Day/month/year)** |
| --- | --- | --- | --- | --- | --- | --- |
| Abatacept | Orencia | Juvenile idiopathic arthritis | List | Clinical and price | None | 22/04/09 |
| Adalimumab | Humira | Hidradenitis suppurativa | List^4^ | Clinical and price | $62,794/QALY | 19/05/16 |
| Adalimumab | Humira | Polyarticular juvenile idiopathic arthritis | List | Clinical only | $60,296/QALY (7-year) / $25,759/QALY (lifetime) | 18/07/13 |
| Agalsidase beta | Fabrazyme | Fabry disease | Do not list | Clinical only | None | 18/05/05 |
| Agalsidase alfa | Replagal | Fabry disease | Do not list | Clinical only | None | 24/11/04 |
| Alglucosidase alfa | Myozyme | Glycogen storage disease (Pompe’s disease) | List | Clinical only | None | 14/06/07 |
| Ambrisentan | Volibris | Idiopathic primary PAH | List | Clinical only | None | 4/02/09 |
| Asfotase alfa | Strensiq | Pediatric onset  hypophosphatasia | List^4^ | Clinical only | $2,698,950/QALY | 23/03/16 |
| Aztreonam | Cayston | CF with chronic pulmonary pseudomonas aeruginosa infection | List | Clinical only | None | 18/07/11 |
| Canakinumab | Ilaris | Systemic juvenile idiopathic arthritis | List^4^ | Clinical only | $824,000/QALY | 17/06/16 |
| Canakinumab | Ilaris | CAPs | Do not list | Clinical only | None | 26/01/11 |
| Clostridium botulinum neurotoxin type A | Xeomin | Blepharospasm | List | Clinical and price | None | 16/12/09 |
| Clostridium botulinum neurotoxin type A | Xeomin | CD | List | Clinical and price | None | 16/12/09 |
| Collagenase clostridium histolyticum | Xiaflex | Dupuytren's contracture with a palpable cord | List^4^ | Clinical and price | None | 27/03/13 |
| Deferasirox | Exjade | Chronic iron overload | List | Clinical and price | $67,595/QALY | 19/04/07 |
| Deferiprone | Ferriprox | Tansfusional iron  overload due to thalassemia | List | Clinical and price | Dominant^5^ | 18/03/16 |
| Denosumab^6^ | Xgeva | Prevention of skeletal-related events due to breast cancer/bone metastases | List^4^ | Clinical and price | >$395,000/QALY | 25/02/16 |
| Denosumab^6^ | Xgeva | Skeletal events due to bone metastases from solid tumors | List^4^ | Clinical only | None | 23/03/16 |
| Dexamethasone intravitreal implant | Ozurdex | Macular edema following central retinal vein occlusion | Do not list | Clinical and price | $21,568/QALY | 25/04/12 |
| Eculizumab | Soliris | Paroxysmal nocturnal hemoglobinuria | Do not list | Clinical and price | $500,000 to $2.4 million/QALY | 19/02/10 |
| Eculizumab | Soliris | Atypical hemolytic uremic syndrome | Do not list | Clinical Only | None | 18/07/13 |
| Elosulfase alfa | Vimizim | Mucopolysaccharidosis IVA | List^4^ | Clinical only | $1,702,127/QALY | 20/05/16 |
| Eltrombopag olamine | Revolade | Adult chronic TP | Do not list | Clinical only | Confidential^7^ | 24/10/11 |
| Everolimus | Afinitor | Renal angiomyolipoma associated with tuberous sclerosis complex | Do not list | Clinical only | None | 25/09/13 |
| Everolimus | Afinitor | Subependymal giant cell astrocytoma associated with tuberous sclerosis complex | Do not list | Clinical only | None | 15/04/15 |
| Evolocumab | Repatha | Heterozygous familial hypercholesterolemia | List^4^ | Clinical and price | $18,457-$34,744/QALY | 19/02/16 |
| Galsulfase^6^ | Naglazyme | Mucopolysaccharidosis VI | List^4^ | Clinical and price | None | 19/02/16 |
| Histrelin acetate | Vantas | Palliative treatment of hormone dependent advanced carcinoma of the prostate | Do not list | Clinical only | None | 26/04/07 |
| Icatibant | Firazyr | Hereditary angioedema | List^4^ | Clinical only | None | 19/12/14 |
| Idursulfase | Elaprase | Mucopolysaccharidosis II (Hunter syndrome) | Do not list | Clinical only | None | 19/12/07 |
| Ivacaftor | Kalydeco | CF with G551D mutation | List^4^ | Clinical and price | $700,000/ QALY | 22/03/13 |
| Lanreotide acetate | Somatuline Autogel | Acromegaly | List | Price only | None | 19/07/07 |
| Laronidase | Aldurazyme | Mucopolysaccharidosis 1 | Do not list | Clinical only | None | 14/07/05 |
| Lomitapide | Juxtapid | Homozygous familial hypercholesterolemia | Do not list | Clinical only | None | 17/04/15 |
| Lumacaftor/  ivacaftor | Orkambi | CF  F508del-CFTR mutation | Do not list | Clinical only | $485,767/QALY | 26/10/16 |
| Macitentan | Opsumit | PAH | List^4^ | Clinical and price | None | 28/01/15 |
| Miglustat | Zavesca | Type 1 Gaucher disease | Do not list | Clinical only | None | 24/11/04 |
| Nintedanib | Ofev | Idiopathic pulmonary  fibrosis | List^4^ | Clinical and price | $248,186/QALY | 15/10/15 |
| Pasireotide diaspartate | Signifor | Cushing’s disease | Do not list | Clinical only | None | 24/02/15 |
| Pegvisomant | Somavert | Acromegaly | Do not list | Clinical only | $137,000/QALY | 2/08/06 |
| Pirfenidone | Esbriet | Idiopathic pulmonary fibrosis | List^4^ | Clinical only | $78,024/QALY | 15/04/15 |
| Plerixafor | Mozobil | Hematopoietic stem cell mobilizer in NHL and MM | Do not list | Clinical only | $19,191/QALY(NHL) $60,835/QALY(MM) | 26/09/12 |
| Posaconazole | Spriafil | Prophylaxis/treatment of invasive aspergillosis | Do not list | Clinical and price | None | 30/01/08 |
| Rifaximin | Zaxine | Overt hepatic encephalopathy | List | Clinical and price | $22,571/QALY | 16/04/15 |
| Riociguat | Adempas | Chronic thromboembolic pulmonary hypertension | List^4^ | Clinical and price | $173,524/QALY | 17/07/14 |
| Rituximab | Rituxan | Granulomatosis with polyangiitis and microscopic polyangiitis | List | Clinical and price | None | 16/08/12 |
| Romiplostim | Nplate | ITP | Do not list | Price only | Confidential^7^ | 27/05/10 |
| Rufinamide | Banzel | Lennox-Gastaut syndrome | List | Clinical and price | $55,715 - $362,127/QALY | 15/03/12 |
| Sapropterin | Kuvan | Phenylketonuria | List^4^ | Clinical and price | $488,182 - $573,314/QALY | 26/10/16 |
| Selexipag | Uptravi | PAH | List^4^ | Clinical only | $187,418/QALY | 26/10/16 |
| Sildenafil citrate | Revatio | PAH | List | Clinical and price | None | 14/02/07 |
| Sodium oxybate | Xyrem | Cataplexy in patients with narcolepsy | Do not list | Price only | $73,096 - $106,607/QALY | 28/01/09 |
| Sodium phenylbutyrate | Pheburane | Urea cycle disorders | List | Clinical only | None | 6/06/16 |
| Somatropin | Genotropin | Growth hormone deficiency | List | Clinical and price | None | 20/12/13 |
| Somatropin | Genotropin | Turner syndrome | List | Clinical and price | None | 20/12/13 |
| Sorafenib | Nexavar | Renal cell carcinoma | Do not list | Price only | None | 28/02/07 |
| Stiripentol | Diacomit | Dravet syndrome | List^4^ | Clinical and price | $50,122/QALY | 16/10/14 |
| Sunitinib | Sutent | Metastatic renal cell carcinoma | Do not list | Clinical and price | $56,000/QALY | 26/04/07 |
| Sunitinib | Sutent | GIST | List | Clinical and price | $80,000/QALY | 28/03/07 |
| Tadalafil | Adcirca | Idiopathic primary PAH | List^4^ | Price only | $70,753/QALY | 15/07/10 |
| Taliglucerase alfa | Elelyso | Gaucher disease | Do not list | Clinical only | None | 28/10/15 |
| Teduglutide | Revestive | Short bowel syndrome | List^4^ | Clinical and price | $1,600,145/QALY | 27/07/16 |
| Tocilizumab | Actemra | Systemic juvenile idiopathic arthritis | List | Clinical only | $69,845/QALY | 19/07/12 |
| Tocilizumab | Actemra | Polyarticular juvenile idiopathic arthritis | List | Clinical and price | None | 19/03/14 |
| Tolvaptan | Jinarc | Autosomal dominant  polycystic kidney disease | Do not list | Clinical only | $244,402/QALY | 24/02/16 |
| Treprostinil | Remodulin | PAH | List | Clinical only | None | 20/07/06 |
| Velaglucerase alfa | Vpriv | Gaucher disease | List^4^ | Clinical and price | None | 25/04/11 |
| Voriconazole | Vfend | Invasive aspergillosis | List | Clinical only | None | 14/04/05 |

^1^Abbreviations are: CDR, Common Drug Review; QALY, quality adjusted life-year; PAH, Pulmonary arterial hypertension; CF, Cystic fibrosis; CAPS, Cryopyrin associated periodic syndromes; CD, Cervical dystonia; TP, Thrombocytopenic purpura; NHL, Non-Hodgkin’s Lymphoma; MM, Multiple Myeloma; ITP, Idiopathic thrombocytopenic purpura; GIST, Gastrointestinal stromal tumor;

^2^Methods used to define reason for recommendation are described in the manuscript

^3^Cost utility ratios are those provided by the manufacturer except for eculizumab with the paroxysmal nocturnal hemoglobinuria indication and asfotase alfa where the ratio was determined by the Common Drug Review

^4^For these recommendations, CDR suggested a conditional substantial reduction in price or a drug cost that should not exceed the drug plan cost of a comparable therapy

^5^Cost utility analysis compared deferiprone to deferasirox: model concluded deferiprone was dominant (i.e., less costly and more effective)

^6^Based on a request for advice from participating drug plans/drug plan submission

^7^Submissions where cost utility ratios were confidential; however, CDR noted that ratios greatly exceeded conventional standards for cost-effectiveness

**Table S2: Comparison of recommendations in Canada (CDR),^1^** Quebec (INESSS), Scotland (SMC), Australia (PBAC) and New Zealand (PHARMAC) for the selected orphan drugs

| **Drug** | **Indication** | **CDR^1^** | **INESSS** | **SMC** | **PBAC** | **PHARMAC^2^** |
| --- | --- | --- | --- | --- | --- | --- |
| Abatacept | Juvenile idiopathic arthritis | Listed | Listed | Listed | Not listed | Not listed |
| Adalimumab | Hidradenitis suppurativa | Listed^3^ | Not listed | Listed | Listed | Not listed |
| Adalimumab | Polyarticular juvenile idiopathic arthritis | Listed | Listed | Listed | Listed | Listed |
| Agalsidase beta | Fabry disease | Not listed | NR | NR | Listed^4^ | Not listed^5^ |
| Agalsidase alfa | Fabry disease | Not listed | NR | NR | Listed^4^ | Not listed^5^ |
| Alglucosidase alfa | Pompe’s disease | Listed | Listed | Not listed | Listed^4^ | Listed |
| Ambrisentan | PAH | Listed | Listed | Listed | Listed | Listed |
| Asfotase alfa | Pediatric onset  hypophosphatasia | Listed^3^ | Not listed | NR | NR | NR |
| Aztreonam | Gram-negative respiratory infection in CF | Listed | Listed | Listed | Listed | NR |
| Canakinumab | CAPS | Not listed | NR | Not listed^6^ | NR | NR |
| Canakinumab | Systemic juvenile idiopathic arthritis | Listed^3^ | NR | Not listed^6^ | Listed | NR |
| Clostridium botulinum neurotoxin type A | Blepharospasm | Listed | Listed | Listed | Listed | NR |
| Clostridium botulinum neurotoxin type A | CD | Listed | Listed | Listed | Listed | NR |
| Collagenase clostridium histolyticum | Dupuytren’s contracture with a palpable cord | Listed^3^ | Not listed | Listed | Listed | NR |
| Deferasirox | Chronic iron overload | Listed | Not listed | Listed | Listed | Listed |
| Deferiprone | Tansfusional iron overload | Listed | NR | NR | NR | Listed |
| Denosumab | Prevention of skeletal-related  events due to breast cancer bone metastases | Listed^3^ | Listed | NR | Listed | NR |
| Denosumab | Skeletal events due to bone metastases from solid tumors | Listed^3^ | NR^7^ | Not listed^6^ | NR | NR |
| Dexamethasone intravitreal implant | Macular edema following retinal vein occlusion | Not listed | Listed | Listed | Listed | Listed |
| Eculizumab | Paroxysmal nocturnal hemoglobinuria | Not listed | Not listed | Not listed | Listed^4^ | Not listed |
| Eculizumab | Atypical hemolytic uremic syndrome | Not listed | NR | Not listed | Listed | NR |
| Elosulfase alfa | Mucopolysaccharidosis IVA | Listed^3^ | Not listed | Not listed | Listed^4^ | NR |
| Eltrombopag olamine | Adult chronic TP | Not listed | Listed | Listed | Listed | Listed |
| Everolimus | Renal angiomyolipoma | Not listed | NR^7^ | Not listed^6^ | NR^8^ | NR |
| Everolimus | Subependymal giant cell  astrocytoma associated with tuberous sclerosis complex | Not listed | NR^7^ | Not listed^6^ | Listed | Listed |
| Evolocumab | Heterozygous familial hypercholesterolemia | Listed^3^ | Listed | Listed | Listed | NR |
| Galsulfase | Mucopolysaccharidosis VI | Listed^3^ | NR | NR | Listed^4^ | Listed |
| Histrelin acetate | Hormone dependent advanced prostate cancer | Not listed | NR | Listed | NR | NR |
| Icatibant | Hereditary angioedema | Listed^3^ | Listed | Listed | Listed | Listed |
| Idursulfase | Mucopolysaccharidosis II (Hunter syndrome) | Not listed | Not listed | Not listed | Listed^4^ | Listed |
| Ivacaftor | CF with G551D Mutation | Listed^3^ | Not listed | Not listed | Listed | Not listed |
| Lanreotide acetate | Acromegaly | Listed | Listed | Not listed^6^ | Listed | Not listed^5^ |
| Laronidase | Mucopolysaccharidosis 1 | Not listed | Not listed | Not listed | Listed^4^ | Listed |
| Lomitapide | Homozygous familial hypercholesterolemia | Not listed | Listed | Not listed^6^ | NR | NR |
| Lumacaftor/ivacaftor | CF F508del-CFTR mutation | Not listed | Not listed | Not listed | Not listed | NR |
| Macitentan | PAH | Listed^3^ | Listed | Listed | Listed | Listed |
| Miglustat | Type 1 Gaucher disease | Not listed | Not listed | Listed | Listed^4^ | Not listed^5^ |
| Nintedanib | Idiopathic pulmonary  fibrosis | Listed^3^ | Listed | Listed | Listed | Not listed^5^ |
| Pasireotide diaspartate | Cushing’s Disease | Not listed | NR | Not listed^6^ | NR | NR |
| Pegvisomant | Acromegaly | Not listed | Not listed | Listed | Listed | Not listed |
| Pirfenidone | Idiopathic pulmonary fibrosis | Listed^3^ | Not listed | Listed | Listed | Listed |
| Plerixafor | NHL and MM | Not listed | Listed | Listed | Listed | Listed |
| Posaconazole | Invasive aspergillosis | Not listed | Listed | Listed | Listed | Listed |
| Rifaximin | Overt hepatic encephalopathy | Listed | Listed | Listed | Listed | Listed |
| Riociguat | Chronic thromboembolic pulmonary hypertension | Listed^3^ | Listed | Listed | Listed | NR |
| Rituximab | Granulomatosis with polyangiitis and microscopic polyangiitis | Listed | NR | Listed | Listed | Listed |
| Romiplostim | ITP | Not listed | Not listed | Listed | Listed | NR |
| Rufinamide | Lennox-Gastaut syndrome | Listed | Listed | Listed | NR | NR |
| Sapropterin | Phenylketonuria | Listed^3^ | Listed | Not listed^6^ | Listed | Not listed^5^ |
| Selexipag | PAH | Listed^3^ | Not listed | Not listed | Not listed | Not listed |
| Sildenafil citrate | PAH | Listed | Listed | Listed | Listed | Listed |
| Sodium oxybate | Cataplexy in patients with narcolepsy | Not listed | Not listed | Not listed | NR | NR |
| Sodium phenylbutyrate | Urea cycle disorders | Listed | Not listed | Listed | NR | Listed |
| Somatropin | Pediatric growth hormone deficiency | Listed | Listed | Listed | Listed | Listed |
| Somatropin | Turner Syndrome | Listed | Listed | Listed | Listed | Listed |
| Sorafenib | Renal cell carcinoma | Not listed | Not listed | Not listed | Listed | Not listed^5^ |
| Stiripentol | Dravet Syndrome | Listed^3^ | Listed | Listed | NR | NR |
| Sunitinib | Metastatic renal cell carcinoma | Not listed | Listed | Not listed | Listed | Listed |
| Sunitinib | GIST | Listed | Listed | Listed | Listed | Listed |
| Tadalafil | PAH | Listed^3^ | Listed | Listed | Listed | NR |
| Taliglucerase | Gaucher Disease | Not listed | Not listed | NR | Listed^4^ | NR |
| Teduglutide | Short bowel syndrome | Listed^3^ | Not listed | Not listed^6^ | NR | NR |
| Tocilizumab | Systemic Juvenile Idiopathic Arthritis | Listed | Listed | Listed | Listed | Listed |
| Tocilizumab | Polyarticular juvenile idiopathic arthritis | Listed | Listed | Listed | Listed | Listed |
| Tolvaptan | Polycystic kidney disease | Not listed | Not listed | Listed | Not listed | NR |
| Treprostinil | PAH | Listed | Listed | NR | Listed | NR |
| Velaglucerase alfa | Gaucher disease | Listed^3^ | Not listed | Listed | Listed^4^ | NR |
| Voriconazole | Invasive aspergillosis | Listed | Listed | Listed | Listed | Listed |
| **Number of drugs reviewed** |  | **68** | **55** | **60** | **55** | **40** |
| **Positive recommendations N (%)** |  | **43 (63.2%)** | **33 (60.0%)** | **38 (63.3%)** | **51 (92.7%)** | **27 (67.5%)** |

^1^Abbreviations are: CDR, Common Drug Review; INESSS, Institut national d’excellence en santé et en services sociaux; SMC, Scottish Medicines Consortium; PBAC, Pharmaceutical Benefits Advisory Committee; PHARMAC, Pharmaceutical Management Agency; NR, not reviewed; PAH, Pulmonary arterial hypertension; CF, Cystic fibrosis; CAPS, Cryopyrin associated periodic syndromes; CD, Cervical dystonia; TP, Thrombocytopenic purpura; NHL, Non-Hodgkin’s lymphoma; MM, Multiple myeloma; ITP, Idiopathic thrombocytopenic purpura; GIST, Gastrointestinal stromal tumour

^2^ Drug status in New Zealand reflects information extracted from the Application Tracker which provides information on pharmaceutical funding applications being considered by PHARMAC

^3^Recommendation from CDR was conditional on a reduction in price or the cost of the drug should not exceed the cost of a comparator

^4^ Drugs funded through the Life Saving Drugs Program in Australia

^5^ Following the PHARMAC review, these drugs were indicated as being “ranked” meaning that the drug has been placed on the list of drugs being considered for funding and when funds are available, it will be considered alongside other medicines on the list. In this table these drugs are indicated as not listed at the time of the review

^6^ SMC issued a not recommended decision in the absence of a submission from the pharmaceutical company

^7^ Not reviewed for this specific indication by INESSS

^8^ Not reviewed for this specific indication by PBAC
